# Melatonin Prevents UVB-Induced Skin Photoaging by Inhibiting Oxidative Damage and MMP Expression through JNK/AP-1 Signaling Pathway in Human Dermal Fibroblasts

**DOI:** 10.3390/life12070950

**Published:** 2022-06-24

**Authors:** Mehtap Yuksel Egrilmez, Semra Kocturk, Sebnem Aktan, Gulgun Oktay, Halil Resmi, Hatice Simsek Keskin, Gul Guner Akdogan, Sebnem Ozkan

**Affiliations:** 1 Department of Molecular Medicine, Institute of Health Sciences, Dokuz Eylul University, Izmir 35340, Turkey; 2 Department of Biochemistry, Faculty of Medicine, Dokuz Eylul University, Izmir 35340, Turkey; semra.kocturk@deu.edu.tr (S.K.); gulgun.oktay@deu.edu.tr (G.O.); halil.resmi@deu.edu.tr (H.R.); akdogan.gul@izmirekonomi.edu.tr (G.G.A.); 3 Department of Dermatological and Venereal Disease, Faculty of Medicine, Dokuz Eylul University, Izmir 35340, Turkey; sebnem.aktan@deu.edu.tr (S.A.); sebnem.ozkan@deu.edu.tr (S.O.); 4 Department of Public Health, Faculty of Medicine, Dokuz Eylul University, Izmir 35340, Turkey; hatice.simsek@deu.edu.tr; 5 Faculty of Medicine, Izmir University of Economics, Izmir 35330, Turkey

**Keywords:** ultraviolet B, melatonin, oxidative damage, JNK pathway, activator protein-1, matrix metalloproteinase

## Abstract

Exposure to ultraviolet (UV) irradiation causes damage to the skin and induces photoaging. UV irradiation stimulates production of reactive oxygen/nitrogen species, which results in activation of epidermal growth factor receptor (EGFR) and mitogen-activated protein kinases (MAPK) in fibroblasts. MAPKs are responsible for activation of activator protein-1 (AP-1), which subsequently upregulates expression of matrix metalloproteinases (MMPs). Melatonin is a potent free radical scavenger which is known to have photoprotective effects. The aim of this study is to investigate the underlying molecular mechanisms for the photoprotective effects of melatonin in UVB-irradiated primary human dermal fibroblasts (HDFs) in terms of EGFR activation, oxidative/nitrosative damage, JNK/AP-1 activation, MMP activities, and the levels of tissue inhibitors of metalloproteinase-1 (TIMP-1) and type I procollagen (PIP-C). In this study, HDFs were pretreated with 1 μM of melatonin and then irradiated with 0.1 J/cm^2^ of UVB. Changes in the molecules were analyzed at different time points. Melatonin inhibited UVB-induced oxidative/nitrosative stress damage by reducing malondialdehyde, the ratio of oxidized/reduced glutathione, and nitrotyrosine. Melatonin downregulated UV-induced activation of EGFR and the JNK/AP-1 signaling pathway. UVB-induced activities of MMP-1 and MMP-3 were decreased and levels of TIMP-1 and PIP-C were increased by melatonin. These findings suggest that melatonin can protect against the adverse effects of UVB radiation by inhibiting MMP-1 and MMP-3 activity and increasing TIMP-1 and PIP-C levels, probably through the suppression of oxidative/nitrosative damage, EGFR, and JNK/AP-1 activation in HDFs.

## 1. Introduction

Exposure to solar ultraviolet (UV) irradiation impacts skin health adversely and causes premature skin aging (photoaging) through multiple molecular pathways [1]. Solar UV radiation at the Earth’s surface is approximately 5–10% UVB (290–320 nm) and 90–95% UVA (320–400 nm) [2]. UVB irradiation, a minor but high-energy component of solar light, induces detrimental effects on the skin. The clinical features of UV-induced photoaging are characterized by fine and coarse wrinkling, mottled hyperpigmentation, and rough skin textures [3]. Extensive dermal connective tissue damage is the prominent hallmark of photoaged skin, resulting in damaged and disorganized collagen fibrils and excessive deposition of abnormal elastic material [4].

Activation of cell surface growth factor and cytokine receptors is one of the earliest cellular responses to UV irradiation. Among these receptors, epidermal growth factor receptor (EGFR) (known as ErbB1 or HER1) is critical in mediating many UV irradiation-induced signal transduction pathways [5,6,7]. EGFR is a single chain transmembrane protein that posseses intrinsic tyrosine kinase activity, thereby catalyzing trans-phosphorylation of specific tyrosine residues in its carboxyl-terminal cytoplasmic domain. These phospho-tyrosines provide binding motifs for effector proteins which initiate the activation of small GTP-binding proteins such as Ras, Rac, and Cdc42, and these proteins in turn activate mitogen-activated protein kinase (MAPK) signaling pathways [8,9]. UV irradiation triggers EGFR tyrosine phosphorylation in skin cells within minutes [10,11].

Exposure to UV irradiation induces oxidative stress via interaction with endogenous absorbing chromophores or molecular oxygen, or alternatively via Rac-activated nicotinamide adenine dinucleotide phosphate (NADPH) oxidase [12,13,14]. This interaction causes production of excess reactive oxygen species (ROS) such as singlet oxygen (^1^O_2_), superoxide anion (O_2_•^−^), hydrogen peroxide (H_2_O_2_), and hydroxyl radical (•OH), as well as reactive nitrogen species (RNS) such as peroxynitrite anion (ONOO-) [15,16,17]. Oxidative modification of catalytic or regulatory cysteine residues of protein-tyrosine phosphatases (PTPs) by UV-induced ROS production results in amplified and/or prolonged tyrosine phosphorylation of EGFR [18,19,20]. Production of ROS ultimately causes cellular lipid and protein peroxidation [21,22,23] and decreases the anti-oxidant defense system by reducing levels of the important antioxidant glutathione (GSH) [24,25]. 

UV activation of EGFR and concomitant production of ROS/RNS leads to activation of MAPK signal transduction pathways, which mediate downstream cellular responses via a cascade of phosphorylation events [6,26,27,28]. MAPKs are a family of serine and tyrosine kinases that includes extracellular signal-regulated kinase (ERK), c-Jun amino-terminal kinase (JNK), and p38 MAPK in mammalian cells [29,30]. The major downstream effector of the MAPK pathways is the activator protein-1 (AP-1), a transcription factor composed of homo- or heterodimeric complexes of Jun and Fos family proteins [31]. UV irradiation increases phosphorylation and protein expression of c-Jun and c-Fos, resulting in active c-Jun:c-Fos AP-1 complexes in human dermal fibroblasts (HDFs) in vitro [32,33,34]. AP-1 regulates transcription of several matrix metalloproteinases (MMPs), which are responsible for degrading extracellular matrix (ECM) proteins that form the skin dermal connective tissue [7,35]. Dermal fibroblasts produce collagen and other ECM proteins in human skin. Type I collagen is the major structural component in the dermal extracellular matrix, providing strength and resiliency to the skin [36]. There is a balance between collagen synthesis and the degradation of matrix collagen by MMPs. This balance is disrupted by UV-induced oxidative stress, which up-regulates the activities of MMP-1 (interstitial collagenase) and MMP-3 (stromelysin-1) in human skin in vivo and in HDFs in vitro [4,33,37,38,39,40]. This increase in the expression of MMPs leads to collagen breakdown, resulting in fragmented and disorganized collagen fibrils [4,33,40,41,42,43]. Loss of balance between MMPs and tissue inhibitors of metalloproteinases (TIMPs) contributes to skin connective tissue damage in photoaging [33,44]. In addition, UV irradiation inhibits the expression of type I procollagen (PIP-C), a precursor of the mature type I collagen, leading PIP-C biosynthesis to increase in response after UV exposure [42]. 

Melatonin (*N*-acetyl-5-methoxytryptamine) was first described as a neuroendocrine secretory product of the pineal gland [45]; it regulates the circadian rhythm in humans [46]. It exerts antioxidant effects as well, acting directly as a free radical scavenger and indirectly by stimulating the production of antioxidant enzymes [47,48,49,50]. Melatonin has anti-inflammatory, immunomodulatory, and anti-tumor capacities [48]. Melatonin actions are mediated through its membrane-bound receptors or through receptor-independent mechanisms [51]. Furthermore, melatonin is synthesized in the skin, which is considered noteworthy for dermatological research [52]. Several studies have shown the photoprotective effects of melatonin against UV-induced skin damage [21,53,54,55,56]. Clinical studies of humans have shown the photoprotective potential of topical treatment of melatonin against UV-induced erythema [57,58]. Melatonin prevents the formation of various reactive free radicals (•OH, O_2_•^−^, ^1^O_2_, H_2_O_2_, ONOO- and peroxyl radical (ROO^.^)) and mediates the reduction of ROS-generated oxidative stress [59,60,61]. Melatonin is metabolized enzymatically or non-enzymatically, and generates a variety of metabolites from its antioxidant cascade. Melatonin metabolites function as potent antioxidants, and have a similar or even better potency in acting as free radical scavengers or inducers of antioxidative enzymes [62,63,64,65]. These metabolites greatly increase the antioxidant capacity of melatonin and provide superiority over other antioxidants, including vitamin C, vitamin E, and glutathione [66]. Due to its hydrophilic and lipophilic structure, melatonin can easily cross cell membranes and reach all cellular compartments [67,68,69], a characteristic which supports its protective effects on intracellular and nuclear fragments against oxidative stress [70]. 

Although the photoprotective effects of melatonin have been documented in several reports, the underlying molecular mechanisms for the photoprotective effects of melatonin in UVB-irradiated HDFs remain to be clarified. In order to better understand the photoprotective properties and molecular mechanisms of melatonin, we further examined UVB-induced signaling pathways from the cell membrane receptor to the nuclear transcription factor. Thus, in the present study, we investigated the effects of melatonin on EGFR activation, the levels of oxidative/nitrosative stress products (such as malondialdehyde (MDA), oxidized/reduced glutathione (GSSG/GSH), and protein nitrotyrosine (NT)), activation of the JNK and AP-1 (c-Jun/c-Fos) transcription factors, the activities of MMPs, and the levels of TIMP-1 and PIP-C in UVB-irradiated HDFs in vitro. 

## 2. Materials and Methods

### 2.1. Chemicals

Melatonin was obtained from Sigma Aldrich (Steinheim, Germany). Dulbecco’s Modified Eagle’s medium (DMEM), fetal calf serum (FCS), penicillin–streptomycin, and phosphate buffered saline (PBS) were purchased from Biochrom (Berlin, Germany). Collagenase was obtained from Sigma Aldrich (Steinheim, Germany) and dispase was purchased from Calbiochem (San Diego, CA, USA).

### 2.2. Skin Biopsies

Ten healthy female volunteers between the ages of 20 and 40 years of age participated in the present study. The skin types of individual participants were evaluated according to the Fitzpatrick classification and assessed as types III–IV by Department of Dermatology, Dokuz Eylul University. This study was approved by the Ethics Committee of Dokuz Eylul University (Approval No: 03/14/03-350, 26 September 2003). All donors provided written informed consent before participation. Skin biopsies of 6–8 mm and full thickness were obtained from sun-protected buttock skin of each subject by punch biopsy. Samples were placed in a sterile plastic canonical tube containing cold culture medium and brought to the laboratory within thirty minutes. Primary adult HDFs were prepared as indicated below. 

### 2.3. Preparation of Primary Adult HDFs 

Punch biopsies were rinsed twice with sterile PBS, minced, and then underwent enzymatic digestion. The tissue was incubated at 37 °C for 1 h in the enzyme mixture (Collagenase (100 U/mL) + Dispase (1.25 U/mL)). The dissociated tissue was resuspended in cell culture medium consisting of DMEM supplemented with 10% FCS (*v/v*), penicillin (100 U/mL), streptomycin (100 mg/mL), amphotericin B (1 μg/mL), and glutamine (1 mM), then transferred to 35 mm dish for the proliferation of fibroblasts from the tissues [71]. The cells were maintained in the cell culture medium mentioned above in a humidified incubator with 5% CO_2_ at 37 °C, and grown to 80% confluence. The fibroblasts were harvested and mixed in a pool for all the experiments. Cells were used between passages three and eight. In all experiments, cells were incubated in the medium with 0.1% FCS (*v/v*).

### 2.4. Study Design 

The cells were divided into four groups: Control group (C), untreated and non-irradiated; Melatonin group (M), only 1 h melatonin treatment; UVB group (UVB), UVB-irradiated; and UVB and Melatonin group (UVB+M), UVB-irradiated after 1 h melatonin pretreatment.

### 2.5. Melatonin Pretreatment 

Melatonin was dissolved in 95% ethanol and prepared as a 0.1 M stock solution, then diluted with cell culture medium to achieve 1 µM of melatonin. The cells were pretreated with melatonin 1 h before UVB exposure. The incubation time was chosen according to a previous study [21]. The control cells were treated with 0.001% final ethanol concentration in cell culture medium corresponding to ethanol concentration in 1 µM of melatonin.

### 2.6. Exposure of Cells to UVB Irradiation 

UVB irradiation was performed using a Waldmann UV181 BL instrument (Waldmann, Germany) with an emission spectrum at 311 ± 5 nm. UV intensity was measured using a Waldmann UV meter (model no. 585 100). After pretreatment with melatonin, the cell culture media were removed and retained for reuse after UVB irradiation. Then, the cells were covered with a thin layer of sterile 1× PBS and irradiated with 0.1 J/cm^2^ of UVB. This dose corresponds to roughly 4-8 minimal erythema doses, which represents a moderately severe sunburn [72]. The UVB dose was adjusted in the UVB irradiation device and the duration of UVB irradiation for this dose was calculated automatically by the instrument. In our study, the irradiation of cells with a UVB dose of 0.1 J/cm^2^ lasted 6 s using the Waldmann UV181 BL instrument. After UVB irradiation, PBS was removed and replaced by the original cell culture medium containing melatonin and the cells were further incubated for the indicated times at 37 °C, 5% CO_2_.

### 2.7. Cell Viability

Cells were treated with melatonin at concentrations of 0.01, 0.1, 1, and 10 µM for 24, 48, and 72 h. These melatonin concentrations were chosen according to previous studies with HDFs [21,54]. The control cells were treated with the highest ethanol concentration (0.01%) which corresponds to the ethanol concentration in 10 µM of melatonin. Cell viability was tested using the Trypan blue exclusion method [73]. Unstained (viable) and stained (nonviable) cells were counted separately, and the percentage of viable cells was calculated.

### 2.8. Measurement of Total EGFR and Tyrosine-Phosphorylated EGFR (EGFR-PY)

Total EGFR and tyrosine 1068 phospho-EGFR in the cell lysates were measured using a commercially available enzyme-linked immunosorbent assay (ELISA) kit according to the manufacturer’s instructions (Mybiosource, San Diego, CA, USA). The results are expressed as EGFR-PY/Total EGFR protein (U/ng).

### 2.9. Determination of MDA and GSSG/GSH Levels

The oxidative damage in the cells was measured by high-performance liquid chromatography (HPLC) in terms of malondialdehyde (MDA), the end product of lipid peroxidation [74]. Cells were collected and lysates prepared. A 40-μL sample was mixed with 100 μL of H_2_O, 20 μL of 2.8 mmol/L BHT in ethanol, 40 μL of 81 g/L sodium dodecyl sulfate, and 600 μL thiobarbituric acid (TBA) consisting of 8 g/L TBA diluted 1:1 with 200 mL/L acetic acid adjusted to pH 3.5 with NaOH. The mixture was incubated at 95 °C for 60 min. After cooling on ice, 1 mL of pyridine–butanol (1:15) mixture was added to the samples and vortexed. The organic phase (supernatant) was separated by centrifugation at 15,000× *g* for 10 min. The supernatants were injected onto a C18 column (150 × 4.6 mm, 5 µm), and the elution was performed at a flow rate of 0.8 mL/min. The fluorescence was measured at excitation and emission wavelengths of 515 and 553 nm, respectively [75]. The concentrations of MDA were determined from the standard curve; MDA levels are expressed as µM per mg protein.

The antioxidant defense system of the cells was determined by HPLC in terms of GSSH/GSG. Cell pellets were resuspended in 0.1 M Tris-HCl buffer (pH 8.5). In order to measure total glutathione (tGSH) levels in the samples, the disulfide bonds of GSSG were reduced by dithiothreitol and the released GSH was derivatized with ortho-phthalaldehyde [76,77]. The injected sample volume was 5 μL and the flow rate was 0.7 mL/min for the C18 column (250 × 4.6 mm, 5 µm). The mobile phase consisted of 50 mM CH_3_COONa and acetonitrile (pH 6.20) (70:30 *v/v*). Detection was carried out at the 340 nm excitation and 420 nm emission wavelengths. The levels of GSSG/GSH were calculated from the standard curve; the GSSG/GSH ratio is expressed as ng per g protein. 

### 2.10. Measurement of NT Levels

The levels of NT in the cell lysates were assessed using Biooxytech enzyme immunoassay (EIA) kit (OxisResearch, Portland, OR, USA) in accordance with the manufacturer’s instructions. Nitrotyrosine concentrations in the samples were calculated using the standard curve, and are expressed as nM per µg protein. 

### 2.11. AP-1 (c-Jun/c-Fos) Transcription Factor Activity Assay

The transcriptional activity of AP-1 (c-Jun/c-Fos) was detected by highly sensitive ELISA-based TransAM AP-1 c-Fos/c-Jun transcription factor assay kits (Active Motif, Carlsbad, CA, USA) according to the manufacturer’s instructions. Nuclear proteins were prepared as previously described [78]. Five micrograms of cell nuclear extract were incubated in oligonucleotide-coated wells for 20 min at room temperature. The plates were washed with PBS three times, and 100 μL of phospho-c-Jun (1:1500) or c-Fos (1:1000) antibody was added to each well and incubated for 1 h. After three washes with PBS, 100 μL of horseradish peroxidase (HRP)-conjugated antibody (1:1000) was added for 1 h. The plates were then incubated with developing solution for 10 min. The reaction was stopped and the absorbance was read at 450 nm using a spectrophotometer.

### 2.12. Western Blotting

Proteins were extracted from the cells using lysis buffer (50 mM Tris-HCl, pH 7.4, 150 mM NaCl, 1 mM EDTA, 1 mM EGTA, 10 µg/mL aprotinin, 10 µg/mL leupeptin, 5 mM phenylmethylsulfonyl fluoride, and 1 mM DTT) containing 1% NP-40. Lysates were centrifuged at 10,000× *g* for 10 min at 4 °C. Protein concentrations were determined using a bicinchoninic acid protein assay (Sigma, Steinheim, Germany) according to the manufacturer’s instructions. Twenty micrograms of protein were separated by electrophoresis using 10% SDS–polyacrylamide gel and then transferred to polyvinylidene fluoride membranes (Amersham, UK). Membranes were subsequently blocked with 1% nonfat dry milk in Tris-buffered saline Tween 20 (TBST) at room temperature and incubated with p-JNK antibody (Cell Signaling Technology, Beverly, MA, USA) with 1% bovine serum albumin in TBST overnight at 4 °C, followed by incubation for 60 min at room temperature with HRP-conjugated secondary antibody. Proteins were visualized by enhanced chemiluminescence (Amersham, UK). 

### 2.13. Measurement of MMP-1, MMP-3, TIMP-1, and PIP-C 

The activity levels of MMP-1 and MMP-3 in the culture supernatants were determined by MMP-1 and MMP-3 activity assay kits (Amersham, UK) according to the recommendations of the manufacturer. Total MMP activity was assessed through the activation of pro-MMP-1 and pro-MMP-3 by preincubation with p-aminophenylmercuric acetate. Active and total levels of MMP-1 and MMP-3 were normalized with the cellular protein concentrations. The levels of active MMP-1 and MMP-3 were then divided by total levels of MMP-1 and MMP-3 to obtain the ratio of active/total MMP-1 and active/total MMP-3.

The levels of TIMP-1 and PIP-C in the culture supernatants were analyzed using ELISA (Mybiosource, San Diego, CA, USA) and EIA (Takara, Kusatsu, Japan) kits, respectively, using the manufacturer’s protocol; TIMP-1 and PIP-C levels are expressed as ng per mg cellular protein.

### 2.14. Statistical Analysis

Three independent experiments were performed using the pooled HDFs of ten healthy donors for each time point in all analyses, with the results expressed as the mean ± standard deviation (SD). Data were analyzed using SPSS 22.0 software (IBM Corp, Chicago, IL, USA). Repeated measures and the difference between groups were analyzed in the model together (GLM, General Linear Model). The repeated-measures ANOVA F test was applied to analyze the difference between repeated measures and between groups. For this analysis, the parametric assumptions of each model were checked. In order to check for parametric assumptions, the normal distribution of the residual squares of the model was analyzed with the Shapiro–Vilk test. In all models, parametric assumption was provided. The homogeneity of the variances in the models was evaluated with Levene’s test. If the GLM-multivariate test result was significant, ANOVA F tests were used to evaluate the differences between repeat measurements and between groups. If repeated and/or intergroup F tests were significant, post hoc paired comparisons were analyzed with the Bonferroni test. In all statistical analyses, *p* < 0.05 was accepted as the statistical significance limit value. 

## 3. Results

### 3.1. The Effect of Melatonin on the Viability of HDFs 

**Figure 1:**
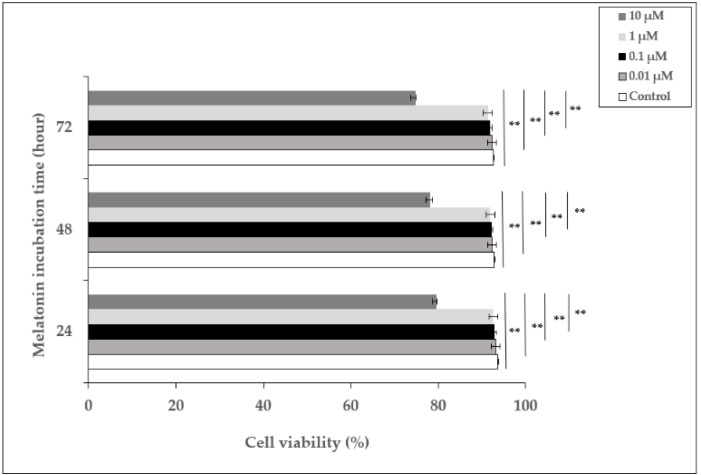
The effect of melatonin on the viability of HDFs. HDFs were treated with various concentrations of melatonin (0.01, 0.1, 1, and 10 µM) for 24, 48, and 72 h. Cell viability was analyzed using a Trypan blue exclusion assay. Results are expressed as the percentage of viable cells and represented as the mean ± SD of three independent experiments of pooled HDFs from ten healthy donors. ** *p* < 0.01.

In order to examine the effect of melatonin, HDFs were incubated with different concentrations of melatonin (0.01–10 µM) for 24, 48, and 72 h, and the percentage of cell viability was determined. The viability of cells incubated with 0.01, 0.1, and 1 µM of melatonin did not show significant differences compared to untreated cells at 24, 48, and 72 h, whereas 10 µM of melatonin decreased cell viability to 79.7%, 78.2%, and 74.8% at 24, 48, and 72 h, respectively (Figure 1). Based on these data, 1 µM of melatonin concentration was chosen as the pretreatment dose for the experiments. 

### 3.2. The Effect of Melatonin on UVB-Induced EGFR Phosphorylation in HDFs

**Figure 2:**
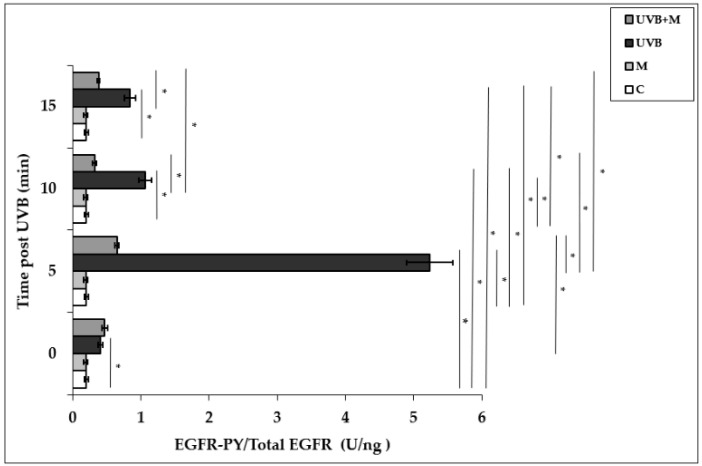
The effect of melatonin on UVB-induced EGFR phosphorylation in HDFs. The cells were pretreated with 1 µM of melatonin for 1 h, further irradiated with 0.1 J/cm^2^ of UVB for 6 s, and then incubated for 0, 5, 10, and 15 min in cell culture medium containing 1 µM of melatonin. Results are expressed as EGFR-PY/Total EGFR protein (U/ng) and represented as mean ± SD of three independent experiments of pooled HDFs of ten healthy donors. C: untreated and non-irradiated control; M: only 1 h melatonin treatment; UVB: UVB-irradiated; UVB+M: UVB-irradiated after 1 h melatonin pretreatment. * *p* < 0.001.

Exposure of HDFs to UVB irradiation resulted in a rapid and significant EGFR phosphorylation in a time-dependent manner. UVB irradiation induced significant increases in EGFR activation at 0, 5, 10, and 15 min (*p* < 0.001). EGFR phosphorylation was increased five-fold at 5 min in UVB-irradiated HDFs compared to non-irradiated cells (5.23 ± 0.34 U/ng) (Figure 2). Pretreatment with melatonin significantly suppressed UVB-induced EGFR phosphorylation at 5, 10, and 15 min (*p* < 0.001) (Figure 2). Our result showed that the highest inhibitory effect of melatonin pretreatment on UVB-induced EGFR activation was observed at 5 min (0.65 ± 0.03 U/ng). 

### 3.3. The Effect of Melatonin on UVB-Induced MDA Levels in HDFs

**Figure 3:**
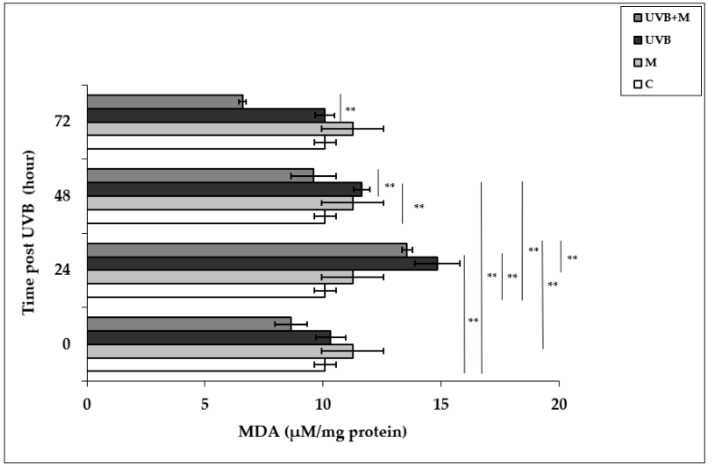
The effect of melatonin on UVB-induced MDA levels in HDFs. The cells were pretreated with 1 µM of melatonin for 1 h, further irradiated with 0.1 J/cm^2^ of UVB for 6 s, and then incubated for 0, 24, 48, and 72 h in cell culture medium containing 1 µM of melatonin. Results are expressed as µM/mg protein and represented as mean ± SD of three independent experiments of pooled HDFs of ten healthy donors. C: untreated and non-irradiated control; M: only 1 h of melatonin treatment; UVB: UVB-irradiated; UVB+M: UVB-irradiated after 1 h melatonin pretreatment. ** *p* < 0.01.

UVB-induced ROS formation causes lipid peroxidation of cell membranes, resulting in oxidative damage to cells [79]. Malondialdehyde, the major end product of lipid degradation, represents a well-established marker for lipid peroxidation [74]. MDA levels were significantly increased in UVB-irradiated HDFs in comparison to non-irradiated cells at 24 and 48 h (14.84 ± 0.96 µM/mg protein and 11.64 ± 0.34 µM/mg protein, respectively; *p* < 0.01) (Figure 3). The highest increase in MDA levels was detected at 24 h after UVB irradiation. Pretreatment with melatonin significantly decreased UVB-induced MDA levels at 24 and 48 h (9.59 ± 0.23 µM/mg protein; *p* < 0.01) (Figure 3).

### 3.4. The Effect of Melatonin on UVB-Induced GSSG/GSH Levels in HDFs

**Figure 4:**
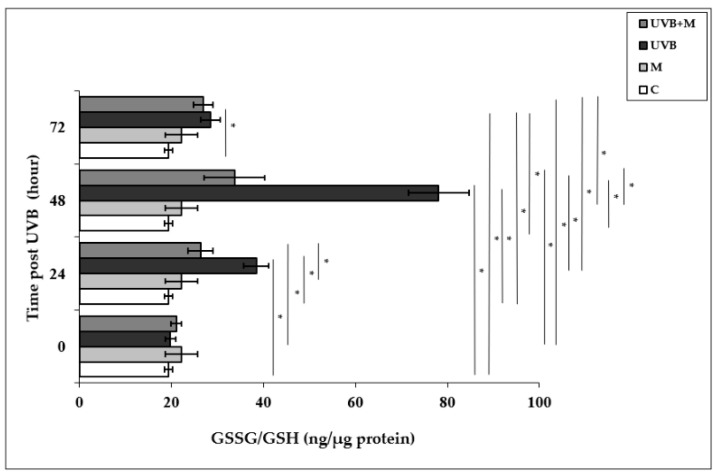
The effect of melatonin on UVB-induced GSSG/GSH levels in HDFs. The cells were pretreated with 1 µM of melatonin for 1 h, further irradiated with 0.1 J/cm^2^ of UVB for 6 s, and then incubated for 0, 24, 48, and 72 h in cell culture medium containing 1 µM of melatonin. Results are expressed as ng/µg protein and represented as mean ± SD of three independent experiments of pooled HDFs of ten healthy donors. C: untreated and non-irradiated control; M: only 1 h of melatonin treatment; UVB: UVB-irradiated; UVB+M: UVB-irradiated after 1 h melatonin pretreatment. * *p* < 0.001.

Glutathione is the most important cellular antioxidant molecule and functions as a reducing agent to neutralize reactivity of free radicals [80]. The GSSG/GSH ratio is used as an important biomarker of the redox balance in a cell, and consequently of cellular oxidative stress. The exposure of skin cells to UVB irradiation is able to cause GSH depletion, and the GSSG/GSH ratio is increased as a result of the oxidation of GSH [24,25]. The GSSG/GSH ratio was significantly higher in UVB-irradiated HDFs compared with non-irradiated cells at 24, 48, and 72 h (38.44 ± 2.74 ng/µg protein, 78.08 ± 6.62 ng/µg protein, and 28.56 ± 2.14 ng/µg protein, respectively; *p* < 0.001) (Figure 4). The highest increase in the GSSG/GSH ratio was found at 48 h after UVB irradiation. Melatonin pretreatment significantly decreased the UVB-increased GSSG/GSH ratio at 24 and 48 h (26.36 ± 1.81 ng/µg protein and 33.71 ± 2.31 ng/µg protein, respectively; *p* < 0.001) (Figure 4).

### 3.5. The Effect of Melatonin on UVB-Induced NT Levels in HDFs

**Figure 5:**
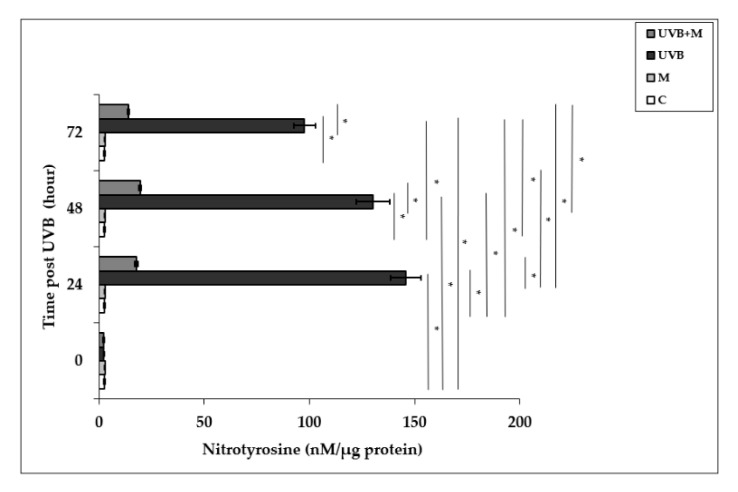
The effect of melatonin on UVB-induced NT levels in HDFs. The cells were pretreated with 1 µM of melatonin for 1 h, further irradiated with 0.1 J/cm^2^ of UVB for 6 s, and then incubated for 0, 24, 48, and 72 h in cell culture medium containing 1 µM of melatonin. Results are expressed as nM/µg protein and represented as mean ± SD of three independent experiments of pooled HDFs of ten healthy donors. C: untreated and non-irradiated control; M: only 1 h of melatonin treatment; UVB: UVB-irradiated; UVB+M: UVB-irradiated after 1 h melatonin pretreatment. * *p* < 0.001.

Free radical-mediated damage to proteins results in the modification of amino acid residues. Nitrotyrosine is formed from nitration of protein-bound and free tyrosine residues by reactive ONOO- molecules [23,81]. Nitrotyrosine is considered to be a relatively specific marker of oxidative damage mediated by the in vivo production of RNS. Exposure to UVB irradiation leads to NT formation. Nitrotyrosine levels were significantly increased in UVB-irradiated HDFs when compared to non-irradiated cells at 24, 48, and 72 h (145.94 ± 7.17 nM/µg protein, 130.31 ± 8.09 nM/µg protein, and 97.71 ± 5.24 nM/µg protein, respectively; *p* < 0.001) (Figure 5). The maximum level was detected at 24 h after UVB exposure. Melatonin pretreatment significantly suppressed UVB-induced NT levels at 24, 48, and 72 h (17.79 ± 0.62 nM/µg protein, 19.54 ± 0.66 nM/µg protein, and 13.95 ± 0.24 nM/µg protein, respectively; *p* < 0.001) (Figure 5).

### 3.6. The Effect of Melatonin on UVB-Induced c-Jun and c-Fos Activation and JNK Phosphorylation in HDFs

**Figure 6:**
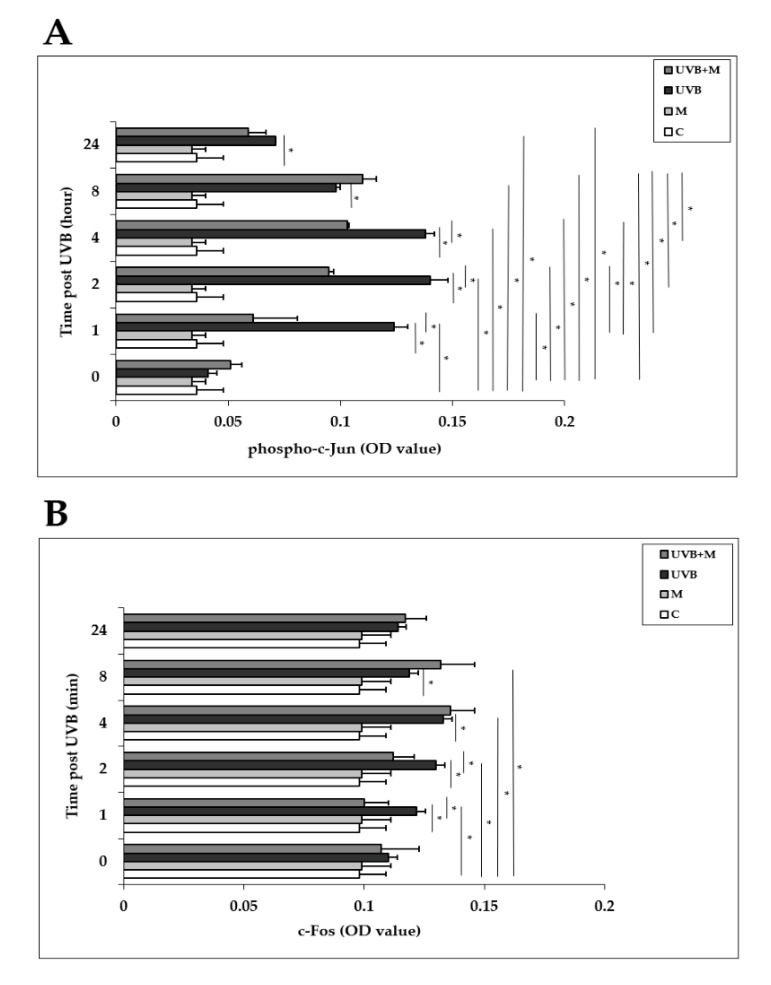
The effect of melatonin on UVB-induced c-Jun and c-Fos activation in HDFs: (**A**) phospho-c-Jun; (**B**) c-Fos. The cells were pretreated with 1 µM of melatonin for 1 h, further irradiated with 0.1 J/cm^2^ of UVB for 6 s, and then incubated for 0, 1, 2, 4, 8, and 24 h in cell culture medium containing 1 µM of melatonin. Results are expressed as OD values measured at 450 nm and are represented as the mean ± SD of three independent experiments of pooled HDFs from ten healthy donors. C: untreated and non-irradiated control; M: only 1 h of melatonin treatment; UVB: UVB-irradiated; UVB+M: UVB-irradiated after 1 h melatonin pretreatment. * *p* < 0.001.

The AP-1 family proteins, which includes c-Jun and c-Fos, are induced in response to UVB exposure in human skin in vivo and in HDFs in vitro [6,32,33,34]. We examined the effects of UVB irradiation with or without melatonin pretreatment on the activation of c-Jun and c-Fos in HDFs at 0, 1, 2, 4, 8, and 24 h. Phosphorylation of c-Jun was significantly increased in UVB-irradiated HDFs compared with non-irradiated cells at 1, 2, 4, 8, and 24 h (*p* < 0.001) (Figure 6A). The highest increases were detected at 1, 2, and 4 h after UVB exposure. Melatonin pretreatment significantly inhibited UVB-increased phospho-c-Jun levels at 1, 2, and 4 h (*p* < 0.001) (Figure 6A). UVB irradiation of HDFs significantly enhanced c-Fos activation compared to non-irradiated cells at 1, 2, 4, and 8 h as well (*p* < 0.001) (Figure 6B). The highest increases were detected at 4 and 8 h after UVB exposure. Pretreatment with melatonin significantly inhibited UVB-increased c-Fos levels at 1 and 2 h (*p* < 0.001) (Figure 6B). 

**Figure 7:**
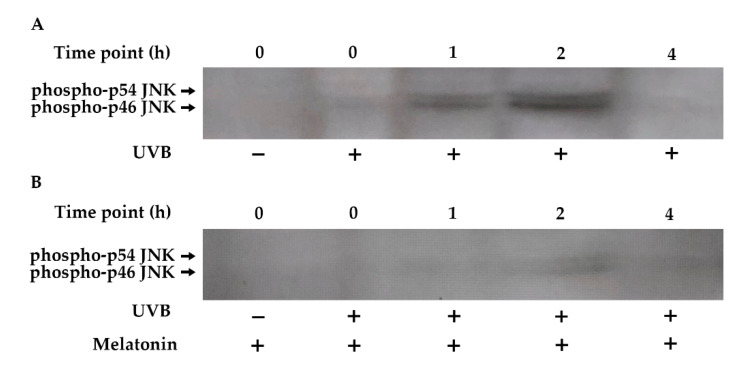
The effect of melatonin on UVB-induced JNK phosphorylation in HDFs. The cells were pretreated with 1 µM of melatonin for 1 h, further irradiated with 0.1 J/cm^2^ of UVB for 6 s, and then incubated for 0, 1, 2, and 4 h in cell culture medium containing 1 µM of melatonin. (**A**) JNK activation in UVB-irradiated groups; (**B**) JNK activation in UVB-irradiated after 1 h of melatonin pretreatment; a representative image from three independent Western blotting experiments is included.

The JNK pathway plays a central role in the UVB-inducible transcription factor AP-1 components (c-Jun and c-Fos) [82,83]. Increased phosphorylation of JNK occurred within 1 h of UVB exposure, remained elevated for 2 h, and returned to baseline levels by 4 h in HDF cells (Figure 7A). Pretreatment with melatonin reduced JNK phosphorylation in UVB-irradiated HDFs (Figure 7B).

### 3.7. The Effect of Melatonin on UVB-Induced MMP-1 and MMP-3 Activities and on TIMP-1 and PIP-C Production 

**Figure 8:**
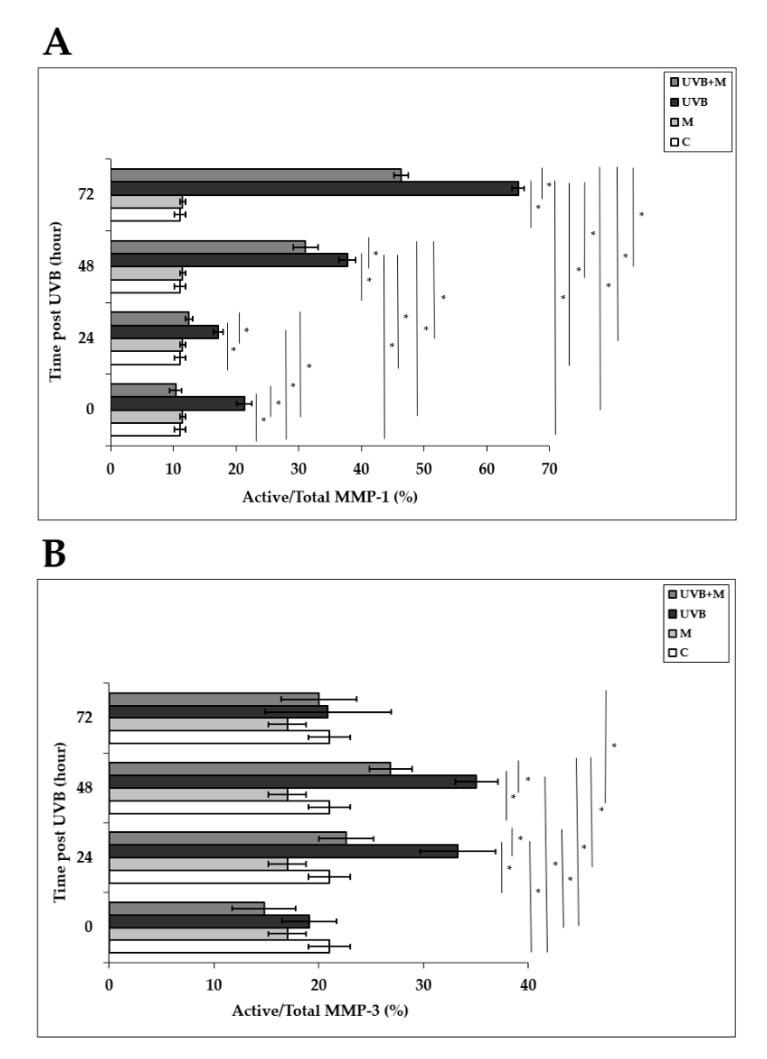
The effect of melatonin on UVB-induced MMP-1 and MMP-3 activity: (**A**) MMP-1; (**B**) MMP-3. The cells were pretreated with 1 µM of melatonin for 1 h, further irradiated with 0.1 J/cm^2^ of UVB for 6 s, and then incubated for 0, 24, 48, and 72 h in cell culture medium containing 1 µM of melatonin. Results are expressed as active/total MMP-1 and active/total MMP-3 and represented as mean ± SD of three independent experiments of pooled HDFs of ten healthy donors. C: untreated and non-irradiated control; M: only 1 h of melatonin treatment; UVB: UVB-irradiated; UVB+M: UVB-irradiated after 1 h melatonin pretreatment. * *p* < 0.001.

Activation of AP-1 following UVB irradiation enhances both mRNA and protein levels and secretion of MMP-1 and MMP-3 in human skin in vivo and in HDFs in vitro [4,33,37,38,39,40]. MMP-1 activity was significantly higher in UVB-irradiated HDFs compared to non-irradiated cells at 0, 24, 48, and 72 h (*p* < 0.001) (Figure 8A). Melatonin pretreatment significantly inhibited MMP-1 activity at 0, 24, 48, and 72 h (*p* < 0.001) (Figure 8A). MMP-3 activity was significantly increased in HDFs exposed to UVB irradiation compared with unexposed cells at 24 and 48 h (*p* < 0.001) (Figure 8B). Pretreatment with melatonin significantly decreased this rise in UVB-induced MMP-3 activity at 24 and 48 h (*p* < 0.001) (Figure 8B). These results demonstrate that melatonin inhibits the activities of multiple MMPs induced by UVB irradiation.

**Figure 9:**
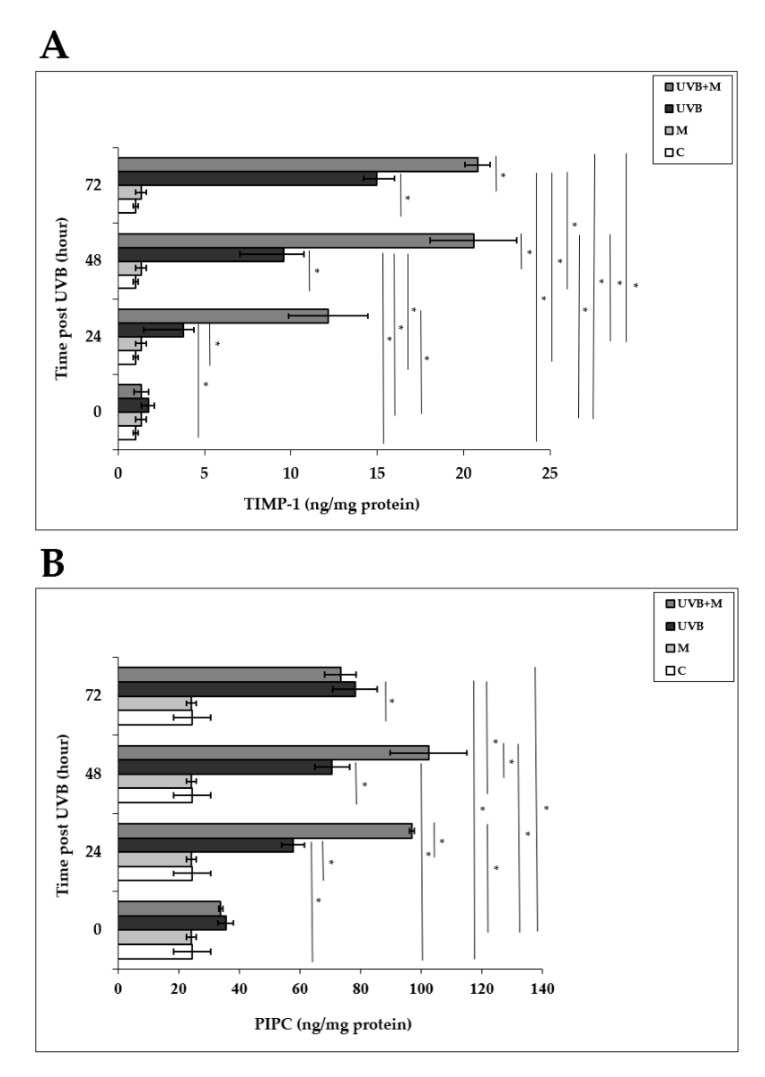
The effect of melatonin on UVB-induced TIMP-1 and PIP-C production: (**A**) TIMP-1; (**B**) PIP-C. The cells were pretreated with 1 µM of melatonin for 1 h, further irradiated with 0.1 J/cm^2^ of UVB for 6 s, and then incubated for 0, 24, 48, and 72 h in cell culture medium containing 1 µM of melatonin. Results are expressed as ng/mg protein and represented as mean ± SD of three independent experiments of pooled HDFs of ten healthy donors. C: untreated and non-irradiated control; M: only 1 h of melatonin treatment; UVB: UVB-irradiated; UVB+M: UVB-irradiated after 1 h melatonin pretreatment. * *p* < 0.001.

Tissue inhibitor of metalloproteinase-1 (TIMP-1) plays an important role in protecting human skin from UVB-induced photodamage [4,33,44,84]. UVB irradiation induced significant increases in TIMP-1 levels in HDFs compared to non-irradiated cells at 24, 48, and 72 h (3.79 ± 0.6 ng/mg protein, 9.57 ± 1.18 ng/mg protein, and 14.97 ± 1.03 ng/mg protein, respectively; *p* < 0.001) (Figure 9A). Melatonin pretreatment significantly increased TIMP-1 levels at 24, 48, and 72 h (12.17 ± 2.3 ng/mg protein, 20.57 ± 2.5 ng/mg protein, and 20.82 ± 0.73 ng/mg protein, respectively; *p* < 0.001) (Figure 9A).

We examined PIP-C production in HDFs at 0, 24, 48, and 72 h after UVB irradiation. PIP-C levels were significantly increased in UVB-irradiated HDFs compared with non-irradiated cells at 24, 48, and 72 h (57.72 ± 3.7 ng/mg protein, 70.65 ± 5.76 ng/mg protein, and 78.19 ± 7.28 ng/mg protein, respectively; *p* < 0.001) (Figure 9B). Pretreatment with melatonin significantly increased UVB-induced PIP-C levels at 24 and 48 h (96.92 ± 0.84 ng/mg protein and 102.38 ± 12.61 ng/mg protein, respectively; *p* < 0.001) (Figure 9B).

## 4. Discussion

It is well-documented that exposure to UVB irradiation induces several pathways to promote skin injury and results in photoaging. Previous findings have provided evidence that the key mechanism by which UVB irradiation initiates molecular responses in human skin is the production of ROS [40,85,86]. 

Dermal fibroblasts are responsible for producing collagen and other ECM components [36]. They are susceptible to UV-induced oxidative damage. Therefore, substances with antioxidative activity could be an effective strategy to protect the skin from the harmful effects of UVB irradiation. Melatonin is an endogenous antioxidant that acts directly as a radical scavenger and indirectly by stimulating production of antioxidant enzymes [47,48,49,50]. Furthermore, previous laboratory and clinical studies have investigated the protective effect of melatonin against UV-induced skin damage [21,53,54,55,56,57,58]. However, the underlying molecular mechanisms for the photoprotective effects of melatonin in UVB-irradiated HDFs remain to be clarified.

In the present study, we set up a UVB irradiation method using primary HDFs. Cells were pretreated with 1 µM of melatonin, further irradiated with 0.1 J/cm^2^ of UVB corresponding to roughly 4–8 minimal erythema doses, and immediately treated again with melatonin for the indicated times. 

Previous studies have shown that UVB irradiation rapidly activates EGFR in skin cells [10,11,87,88]. In agreement with this, we observed EGFR activation in HDFs within minutes following UVB irradiation. Pretreatment with melatonin significantly suppressed EGFR activation at early time points. UVB-induced production of ROS is thought to play a central role in initiating EGFR phosphorylation. Inactivation of the cytoplasmic PTPs by ROS results in accumulation of phosphorylated tyrosine residues on EGFR [18,19,20]. The ability of melatonin to inhibit EGFR phosphorylation might be the result of preventing oxidation of cysteine residues in PTPs with its direct scavenging capacity on multiple radicals, thereby retaining the balance between the EGFR tyrosine kinase and tyrosine phosphatase activities after UVB irradiation. Our results suggest that the inhibition of UVB-induced EGFR activation is involved in the photoprotective effects of melatonin. 

Lipid components in the membranes are highly susceptible to UVB irradiation damage. Increased levels of oxidative stress following UVB exposure causes lipid peroxidation and consequently leads to the formation of lipid peroxidation products. Malondialdehyde is the end product of lipid peroxidation, and is used as a marker of oxidative damage [74]. A previous study has revealed that MDA levels in HDFs increase at 24 h following UVB irradiation and that melatonin inhibits UVB-induced increases in MDA levels [21]. We assessed MDA levels 24, 48, and 72 h after UVB irradiation and found that MDA levels were increased in UVB-irradiated HDFs at 24 and 48 h. Melatonin pretreatment inhibited MDA levels at 48 h. Our data confirm previous reports showing the inhibitory effect of melatonin on MDA levels [21]. Glutathione is the major antioxidant which regulates cellular redox homeostasis, has cytoprotective effects against oxidative damage via direct scavenging of ROS and lipid peroxides, and acts as a cofactor for several antioxidant enzymes [89,90]. An increased GSSG/GSH ratio is considered indicative of oxidative stress. UVB irradiation has been shown to cause GSH depletion in human foreskin fibroblasts and HaCaT keratinocytes in vitro [22,91,92]. As previously reported, UVB irradiation increased GSSG/GSH ratio in HDFs at all examined time points in our study. The result of our experiments showed that melatonin pretreatment markedly decreased UVB-increased GSSG/GSH ratio at 24 and 48 h, suggesting that melatonin plays an important role in maintaining glutathione homeostasis. Nitration of tyrosine residues by ONOO- can reduce tyrosine phosphorylation in tyrosine kinases and thereby interfere with signal transduction pathways involving tyrosine phosphorylation, such as EGFR signaling [23,93]. A low dose of UVB (0.05 J/cm^2^) irradiation has been shown to result in tyrosine nitration of claudin-1 in HaCaT cells [94]. Our results demonstrate that NT levels increased within 24 h and remained elevated for 72 h following UVB irradiation. Pretreatment with melatonin significantly suppressed UVB-induced increases in NT levels at all examined time points. Our results indicate that melatonin at a concentration of 1 µM protected HDFs against UVB-induced oxidative damage by enhancing the GSSG/GSH ratio, thereby reducing lipid peroxidation and protein nitration. Our findings support the previous study of Izykowska et al., who demonstrated a preventive effect of 1 µM of melatonin on the viability of HDFs irradiated with UVB doses of 30 mJ/cm^2^ and 60 mJ/cm^2^ [54]. Another study by Ryoo et al. has reported that melatonin at concentrations of 1 nM and 100 nM increased survival of HDFs exposed to a UVB intensity of 140 mJ/cm^2^ [21]. The difference in melatonin concentrations between their study and ours may be due to the donor ages of the cells, as Ryoo et al. used HDFs isolated from children with mean age of 5 years.

Activation of EGFR by UVB exposure leads to activation of the AP-1 family members c-Jun and c-Fos in HDFs in vitro [32,33,34], and UVB-induced ROS/RNS contribute to AP-1 activation as well. Consistent with previous observations, UVB irradiation induced c-Jun phosphorylation and c-Fos activation in HDFs [32,33,39]. The level of phospho-c-Jun was sustained for 24 h following UVB irradiation. In contrast to our results, Fisher et al. found in their in vivo study that while exposure of human skin to UVB irradiation increased c-Jun activation, it did not alter c-Fos [6]. This could be due to differences between in vitro and in vivo experimental conditions. Our study showed that pretreatment with melatonin markedly inhibited UVB-increased levels of phospho-c-Jun and c-Fos. The inhibitory effect of melatonin was more prominent in phospho-c-Jun levels. We further observed that UVB irradiation induced JNK phosphorylation, and melatonin pretreatment reduced JNK phosphorylation in HDFs. These data suggest that the JNK/AP-1 signaling pathway might underlie the inhibitory activities of melatonin against UVB-induced oxidative damage in HDFs. 

UVB-induced skin photoaging is primarily characterized by coarse wrinkling, rough textures, and loss of elasticity [35]. Previous evidence has shown that AP-1 activation due to UVB exposure upregulates expression of MMP-1 and MMP-3 in human skin in vivo and in HDFs in vitro [4,33,37,38,39,40,95]. UVB-induced production of MMP-1 and MMP-3 causes degradation of ECM components, including collagen and elastin fibers, in the dermis, contributing to wrinkle formation. In agreement with previous studies, the activities of MMP-1 and MMP-3 were increased after UVB irradiation, and melatonin pretreatment significantly decreased the UVB-induced activity of both MMP-1 and MMP-3. Slomonski et al. have shown that 1 mM of melatonin can inhibit mRNA levels of MMP-1 and MMP-3 in HaCaT keratinocytes irradiated with a UVB dose of 30 mJ/cm^2^ [96], and Park et al. have demonstrated that melatonin at concentrations of 1 and 2 mM can inhibit MMP-1 mRNA and protein levels in HaCaT keratinocytes irradiated with a UVB dose of 30 mJ/cm^2^ [56]. However, our results show that a lower concentration of melatonin (1 µM) inhibited both MMP-1 and MMP-3 activity in HDFs exposed to a higher dose of UVB exposure (0.1 J/cm^2^). This may be explained by the direct scavenging capacity of melatonin and its metabolites and their indirect action on the production of antioxidant enzymes in HDFs. In addition, the ability of melatonin to interact with intracellular organelles such as mitochondria and nuclei might contribute to its antioxidant effects [97]. Enhancement of TIMP-1 has been shown to protect against photodamage by inhibiting UVB-induced collagen degradation in vivo [44]. Our in vitro study showed that UVB irradiation induced increases in TIMP-1 levels and pretreatment with melatonin was able to increase TIMP-1 levels in HDFs. Owing to the inhibition of MMP-1 and MMP-3 activities and the increase in TIMP-1 levels, we further assessed the effect of melatonin on PIP-C levels. Fisher et al. have revealed that PIP-C mRNA and protein expression levels were reduced within 8 h following UV (UVB/UVA2) irradiation of human skin in vivo, then increased, leading to new synthesis of mature collagen [42]. We found that PIP-C levels were increased in UVB-irradiated HDFs and that pretreatment with melatonin significantly increased UVB-induced PIP-C levels in HDFs. These findings support the concept that melatonin protects against UVB-induced collagen breakdown by upregulating PIP-C levels, decreasing MMP-1 and MMP-3 activity, increasing TIMP-1 expression, and suppressing the activation of the JNK/AP-1 signaling pathway.

Serum nighttime concentrations of melatonin vary between 100 and 150 pg/mL (~0.43–0.65 nM) in humans. During daylight, serum levels are low (5 pg/mL or less) [98]. Clinical studies have shown that plasma melatonin concentrations increase by 10 to 100 times higher than the physiological nocturnal peak within the hour following oral intake of melatonin (i.e., 0.1 to 10 mg) [99,100,101,102,103]. The time to maximal serum concentration was 50 min and the elimination half-life was approximately 45 min following oral administration of melatonin. The bioavailability of oral melatonin ranged from 9–33% [99,100,101,102,103]. It might therefore be anticipated that maximum serum peak melatonin levels will not reach far beyond 100 nM even upon oral melatonin intake, which is ~200 times higher than endogenous peak levels. Our in vitro concentration of 1 µM melatonin is quite a low dose to reach serum nighttime peak levels. As bioavailability of oral melatonin administration is low as well, mainly due to the first-pass mechanism in the liver, the amount reaching the skin is quite limited. Topical administration of melatonin would therefore be a very good option to protect against photoaging. Clinical studies in humans have shown the photoprotective potential of topical treatment of melatonin against UV-induced erythema [57,58]. Topically-applied ethanol solutions and cream of melatonin have been found to penetrate through the skin [104,105]. Both formulas increased serum levels of melatonin, which lasted 8 h and 24 h after application, respectively. These findings confirm the clinical potential of topical melatonin as a photoprotector. Endogenous skin melatonin production together with topically-administered exogenous melatonin might be a promising approach against photoaging. However, the efficacy of topically-applied melatonin require further evaluation in future clinical trials.

## 5. Conclusions

**Figure 10:**
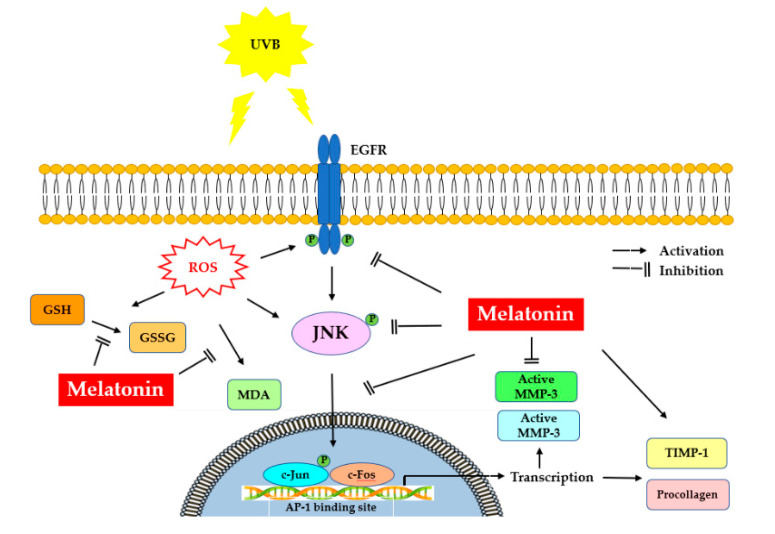
Representation of melatonin’s effects on UVB-induced skin photoaging. UV, ultraviolet; EGFR: epidermal growth factor receptor; ROS: reactive oxygen species; MDA, malondialdehyde; GSSG/GSH, oxidized/reduced glutathione; JNK, c-Jun amino-terminal kinase; AP-1, activator protein-1; MMP-1, matrix metalloproteinase-1; MMP-3, matrix metalloproteinase-3; TIMP-1, tissue inhibitor of metalloproteinase-1; PIPC, type I procollagen.

It is noteworthy that protection of the fibroblasts in the skin from photoinduced damage is of fundamental importance. Our study demonstrates the underlying mechanisms of protection that melatonin can provide to HDFs against UVB-induced damage. Melatonin suppressed UV-induced activation of EGFR, JNK, c-Jun and c-Fos signaling pathways in HDFs, probably mediated through the inhibition of oxidative/nitrosative damage induced by UVB. Furthermore, melatonin enhanced PIP-C levels by inhibiting MMP-1 and MMP-3 activity and increasing TIMP-1 levels, indicating the photoprotective effect of melatonin in UV-irradiated HDFs. Figure 10 summarizes the possible protective mechanisms of melatonin against photoaging in dermal fibroblasts. Although further investigations are needed, these findings suggest the potential of melatonin as a photoprotective agent which can be used for the attenuation and improvement of photoaging.

## Data Availability

The data presented in this study are available within the article.
